# The complex impact of auditory error direction, magnitude, and exposure on corrective responses

**DOI:** 10.3389/fnhum.2025.1729850

**Published:** 2026-01-27

**Authors:** Anuradha J. Sreedhar, Ayoub Daliri

**Affiliations:** College of Health Solutions, Arizona State University, Phoenix, AZ, United States

**Keywords:** adaptive, corrective, error direction, error exposure, speech

## Abstract

**Purpose:**

When encountering errors, the brain produces within-production corrective responses to compensate for the errors. The brain also develops adaptive responses to ensure the accuracy of future productions. This study examined the impact of the number of exposures to errors with different characteristics on corrective and adaptive responses.

**Methods:**

We designed a compensation paradigm to apply three perturbation magnitudes in two directions within the two-dimensional formant space: (1) a concurrent increase in the first formant and decrease in the second formant, acoustically shifting /ɛ/ toward /æ/ (ɛ-to-æ perturbations), and (2) a concurrent increase in the first formant and increase in the second formant, shifting /ɛ/ toward the outside of the vowel space (ɛ-to-out perturbations). To calculate corrective responses in perturbed trials, we determined the difference between formants in the late (300–400 ms) and early (0–100 ms) intervals of the vowel. We estimated adaptive responses in unperturbed trials by calculating formant changes in the early interval of the post-perturbation trial relative to the early interval of the pre-perturbation trial. We examined the responses in the first and second halves of trials in each condition to determine the effect of exposure to perturbations.

**Results:**

We found that (1) corrective responses to ɛ-to-æ perturbations were larger and proportionally larger for small perturbations in the first half of perturbed trials, (2) adaptive responses were measurable after exposure to a single perturbation, and (3) strikingly, both corrective and adaptive response directions were generally in the participant-specific ɛ-ɪ direction, regardless of the perturbation direction. This preferred response direction was in the opposite direction of the ɛ-to-æ perturbations but was perpendicular to the ɛ-to-out perturbations.

**Conclusion:**

Our findings suggest that the brain responds more to small and natural-sounding errors (ɛ-to-æ perturbations) than large and unnatural errors (ɛ-to-out perturbations). The brain’s biases in selecting movements may influence error compensation, leading to responses that prioritize preferred movements even when they do not fully correct errors (e.g., responding in the ɛ-ɪ direction for ɛ-to-out perturbations). Finally, as the brain encounters more unexpected errors, it may evaluate them differently and respond less to them.

## Introduction

We often make errors (e.g., misarticulate words) during speaking, but we usually recognize and correct them during or after production (Perkell, 2012). The ability to detect and correct errors is important for maintaining accurate speech production and avoiding communication breakdowns. This ability is vital when we are learning to produce speech during childhood or to produce a new language (Guenther, 2016). Additionally, many speech disorders negatively impact the speakers’ error detection and correction ability, leading to inaccurate or unintelligible speech (Basciano et al., 2025; Behroozmand et al., 2025; Daliri et al., 2025; Daliri and Max, 2018; Nejati et al., 2025; Parrell et al., 2017; Stepp et al., 2017; Terband et al., 2014). Treatments for speech disorders aim to improve the speaker’s speech by having the speaker practice target sounds trial by trial, with strong emphasis on error detection and correction (for a review, see Maas et al., 2008). Therefore, understanding error-related processes is of paramount importance because these processes are crucial for learning to produce speech during childhood, maintaining accurate speech throughout adulthood, and improving speech in individuals with speech disorders.

When the brain plans to produce a speech sound, it prepares a set of motor commands to generate the speech movements that produce the target sound. Through years of experience, the brain develops internal models that associate motor commands with their sensory consequences (Krakauer et al., 2019). During planning, the brain relies on its internal models to predict the auditory consequences of the motor commands (McNamee and Wolpert, 2019). Using such predictions, the brain optimizes motor commands and prepares the sensory system for speech monitoring (Guenther, 2016; Shadmehr and Mussa-Ivaldi, 2012). After the brain starts producing the movements, the auditory consequences of those movements become available, and the brain begins comparing them with the predicted auditory consequences of the movements (Guenther, 2016; Houde and Nagarajan, 2011; Parrell and Houde, 2019). In response to potential mismatches between the prediction and feedback (i.e., prediction errors), the brain generates corrective responses to reduce the errors. Corrective responses begin within 100–200 ms after receiving the error through auditory feedback; thus, they are typically used as behavioral measures of the feedback-dependent processes that the brain uses to correct errors in its movements (e.g., Tourville et al., 2008). Additionally, the brain relies on the errors to adjust (i.e., adapt) its motor commands so that future attempts would more accurately produce the target sound. Because adaptive responses gradually develop over successive exposures to errors, they are typically used as behavioral measures of feedforward processes that the brain uses to prepare, optimize, and modify motor commands (e.g., Houde and Jordan, 1998). Although the corrective and adaptive responses rely on prediction errors, their underlying processes differ in their sensitivities to errors (i.e., assigning different weights to errors when responding) (Daliri, 2021; Merrikhi et al., 2025), have different underlying neural processes (Guenther, 2016), and function somewhat independently (e.g., Lester-Smith et al., 2020).

A common approach to studying feedback-dependent processes is to induce auditory errors during speech via near-real-time auditory feedback perturbations in randomly selected trials, with unperturbed trials before and after each perturbed trial (i.e., compensation paradigm). Using compensation paradigms, studies have measured corrective responses to perturbations of the fundamental frequency (e.g., Burnett et al., 1997), formants (first formant: F1, second formant: F2, or both F1 and F2) (e.g., Purcell and Munhall, 2006), timing (e.g., Cai et al., 2014), and intensity (e.g., Heinks-Maldonado and Houde, 2005). Recently, we examined changes in corrective responses to formant perturbations with different magnitudes (0.5, 1, and 1.5 of the ɛ–æ distance) and directions (concurrent F1 and F2 perturbations along the ɛ–æ line in the F1-F2 coordinates and F1 perturbation only) (Daliri et al., 2020). We found that (1) corrective responses proportionally decreased as the perturbation magnitude increased, (2) corrective responses to F1-F2 perturbations were generally larger than responses to F1 perturbations, and (3) regardless of the direction of the perturbations, corrective responses were along the ɛ-ɪ line (i.e., participants changed both F1 and F2 to correct for F1-F2 or F1 perturbations). Because both F1-F2 and F1 perturbations had similar perceptual consequences (i.e., formants of /ɛ/ were perturbed toward formants of /æ/), it remained unclear whether participants perceived similar auditory errors and thus generated corrective responses in the opposite direction of the perceived error toward /ɪ/. To address this question, the first objective of the current study was to determine the direction of corrective responses when auditory perturbations are orthogonally oriented in the two-dimensional F1-F2 coordinates, thereby maximally reducing their perceptual similarity.

In the compensation paradigm, perturbations are applied in randomly selected trials, with unperturbed trials before and after each perturbed trial. This procedure minimizes potential changes in feedforward processes and isolates the contributions of feedback-dependent processes (Daliri, 2021; Daliri et al., 2020; Merrikhi et al., 2025). Compensation paradigms differ from commonly used “adaptation” paradigms, in which participants experience perturbations across successive trials, leading to the development of adaptive responses (i.e., changes in feedforward processes). In other words, in adaptation paradigms, participants develop an adaptive response after each exposure to a perturbation, and this response becomes larger as exposure continues (though it plateaus after a certain number of exposures). Thus, one could argue that, in a compensation paradigm, participants may also develop a small adaptive response after exposure to a single perturbation–commonly called “one-shot learning” (Hantzsch et al., 2022; Smith and Shadmehr, 2004). In our previous study (Daliri et al., 2020), we compared formants in the first 100 ms of an unperturbed trial before a perturbed trial with those in the first 100 ms of an unperturbed trial after the perturbed trial to examine adaptive responses to the perturbation. We did not find statistically significant adaptive responses. Similarly, Parrell et al. (2017) examined changes in F1 in the first 50 ms of the vowel of post-perturbation trials and did not find significant F1 adaptive responses. However, by pooling data from six studies (a total of 131 participants), Hantzsch et al. (2022) showed that participants develop small but measurable adaptive responses after experiencing one perturbed trial. Thus, in a compensation paradigm, each exposure to perturbations can lead to changes in internal models and, consequently, in prediction signals. Because both adaptive and corrective responses depend on prediction errors, these responses may gradually change over exposure to perturbed trials. This speculation is supported by evidence from our previous study (Daliri, 2021), where we determined the contributions of the feedback and feedforward systems using an adaptation paradigm. In that study, we used changes at early time points in the vowel (i.e., adaptive responses) as a measure of the feedforward system and changes at late time points in the vowel as a measure of the combined feedforward and feedback systems. At early time points, auditory feedback is unavailable, and speech output is primarily controlled by the feedforward control system. However, the combined feedforward and feedback-dependent processes influence the speech output at late time points. We used the difference between responses at late and early time points as a measure of the feedback-dependent processes (i.e., corrective responses). We found that (1) adaptive responses gradually increased with more exposure to perturbations and reached a plateau after several trials, and (2) corrective responses were the largest in the first trial with auditory perturbations and decreased with more exposure to perturbations. Therefore, the second objective of the current study was to determine whether corrective responses in perturbed trials and adaptive responses in unperturbed trials of a compensation paradigm change with more exposure to perturbed trials.

In the present study, we designed a compensation paradigm to examine the impact of perturbation magnitudes, perturbation directions, and the extent of exposure to perturbations on corrective responses in perturbed trials (as a measure of the feedback-dependent system) and adaptive responses in unperturbed trials (as a measure of the feedforward system). Similar to our previous study (Daliri et al., 2020), we applied three perturbation magnitudes: 0.5, 1.0, and 1.5 times participant-specific ɛ–æ distance. The direction of the perturbation in each perturbed trial was (1) a concurrent F1-increase and F2-decrease in the F1-F2 coordinates, shifting the participant’s /ɛ/ toward /æ/ (ɛ-to-æ perturbations), or (2) a concurrent F1-increase and F2-increase in the F1-F2 coordinates, shifting the participant’s /ɛ/ along the perpendicular line relative to the participant-specific ɛ–æ line (toward the outside of the vowel space; ɛ-to-out perturbations). We measured the responses in the first and second halves of perturbed trials to examine the extent of exposure to perturbations. We measured adaptive responses based on formants in the first 100 ms of the vowel and corrective responses based on changes in formants in 300–400 ms of the vowel relative to formants in 0–100 ms of the vowel. We predicted that participants’ responses would be in the ɛ-ɪ direction for ɛ-to-æ perturbations. Additionally, we hypothesized that responses would be (1) proportionally larger for smaller perturbation magnitudes, (2) larger for ɛ-to-æ perturbations, and (3) larger for the first half of the perturbed trials.

## Materials and methods

### Participants

We recruited 30 participants for this study (9 males; *M*_*age*_ = 21.3 years, SD_*age*_ = 2.5 years; all right-handed). All participants met the following eligibility criteria: (1) did not have a history of speech-language-hearing, neurological, and psychological disorders; (2) were not taking medications that influence the central nervous system; (3) were native speakers of American English; (4) had normal binaural hearing threshold as measured by a standard pure-tone hearing screening (≤20 dB HL at octave frequencies ranging from 250 to 8000 Hz) (ASHA, 1997). All participants signed a written consent form before the experiment, and the experimental protocols were approved by Arizona State University’s Institutional Review Board.

### Apparatus

The experiment was conducted in a double-walled sound-attenuating booth. The participants were seated in a chair facing a computer monitor on which the experimental stimuli were displayed. A microphone was placed at ∼15 cm from the participant’s mouth at a ∼45° angle. The microphone (SM58, Shure) recorded the participant’s speech, and the input signal was then amplified (TubeOpto 8, ART) and sent to a computer via an external audio interface (8pre USB, MOTU). Outside the sound booth, the computer recorded the speech signal, manipulated the auditory feedback, and presented target stimuli (words and sentences). The computer output was sent to the audio interface, amplified (S-phone, Samson Technologies), and sent to the participants via insert earphones (ER2, Etymotic Research). The entire input-to-output loop (microphone to insert earphones) was calibrated before each experiment to ensure the output signal was 5 dB higher than the input signal. Similar to our previous studies (Chao et al., 2019; Chao and Daliri, 2023; Daliri and Dittman, 2019), we used Audapter for real-time formant tracking and formant perturbations. Audapter is implemented in C++ to efficiently process and manipulate speech signals in near real-time (Cai, 2015). Audapter combines Linear Predictive Coding (LPC) and dynamic programming to track formant frequencies. For the current study, we used the following parameters to program Audapter: LPC order of 17 for males and 15 for females; sampling rate of 48000 Hz; downsampling factor of 3; frame length of 32 samples; processing delay of 5 frames. Using a digital audio recorder (DR-680MKII, Tascam), we simultaneously recorded the input (microphone) and output signals (earphones) to determine the total input-output delay (Kim et al., 2020). This combination of parameters resulted in an input-output delay of ∼18 ms.

### Procedure

The experiment was conducted in two sessions, with a minimum of 7 days between sessions. To be consistent with the design of our previous study (Daliri et al., 2020), the experimental procedure across the two sessions was identical, except that participants received perturbations in different directions. To eliminate order effects, we counterbalanced the two perturbation directions (ɛ-to-æ and ɛ-to-out ) across participants. Half of the participants experienced the ɛ-to-æ perturbations in Session 1, followed by ɛ-to-out perturbations in Session 2, while the other half received the reverse order. Each session included several experimental blocks, with brief breaks between them. The following experimental blocks were included in each session: (1) one block of a training task, (2) one block of a pre-test task, and (3) seven blocks of a compensation task.

#### Training task

The training task was the first block that participants completed in each session. This block lasted for 2 min. The purpose of the training task was to (1) train participants to produce the consonant-vowel-consonant target words at a desired intensity (72–82 dB SPL) and duration (450–650 ms) and (2) familiarize participants with reading the target words and hearing themselves through the insert earphones. Each trial began with the presentation of a target word (in black on a gray background) for 2.5 s, followed by a blank page for 1–1.5 s. Participants produced ten repetitions of /hɛp/, /hɛd/, and /hɛk/ (in random order) and received visual feedback regarding the intensity and duration of their vowels after each production. The visual feedback was presented as green (within the range), blue (below the range), or red (above the range) bars at the top (for duration) and bottom (for intensity) of the screen during the blank-page phase of each trial.

#### Pre-test task

The pre-test task was similar to the training task and lasted about 5 min. In this task, we provided visual feedback about the duration and intensity of the vowels when they were outside the desired ranges. We designed the pre-test task to determine participant-specific vowel configurations for front vowels /ɪ/, /ɛ/, and /æ/. Participants produced 25 repetitions of /hɪp/, /hɛp/, and /hæp/ in a random order. Upon the completion of the task, we extracted the average formant values (estimated by Audapter) for each production and visually inspected the vowel distributions in the F1-F2 coordinates. We then used the formant values to calculate (1) vowel centroids (median formant values) of /ɪ/, /ɛ/, and /æ/, (2) ɛ–æ distance, and (3) ɛ–æ angle. We used the formant values of the ɛ-centroid as initial values for Audapter’s formant tracking and the ɛ–æ distance and angle to define participant-specific formant perturbation configurations in the compensation task.

#### Compensation task

**FIGURE 1:**
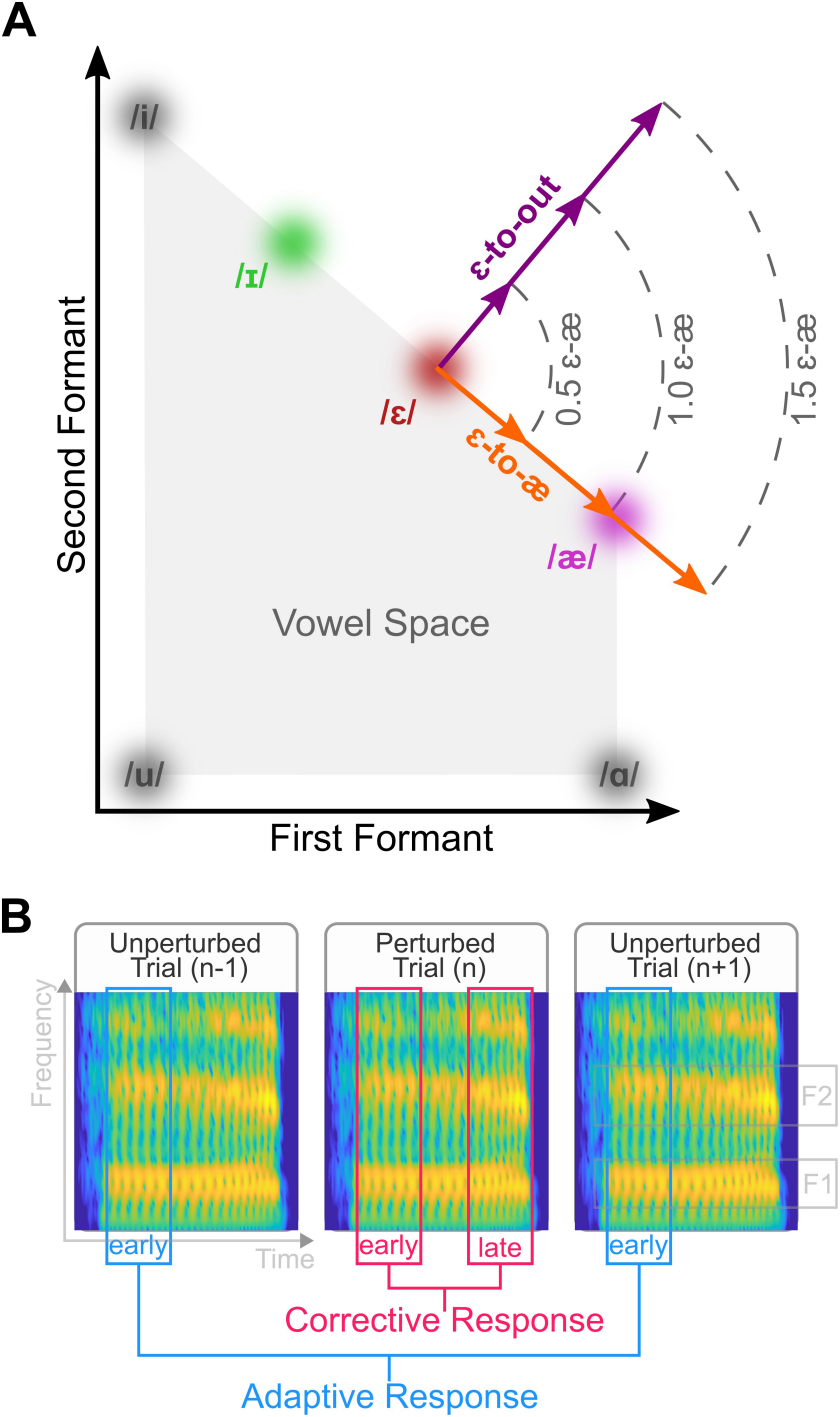
**(A)** We applied participant-specific perturbations in two directions: a concurrent F1-increase and F2-decrease in the F1-F2 coordinates, shifting /ɛ/ toward /æ/ (ɛ-to-æ perturbations), and (2) a concurrent F1-increase and F2-increase in the F1-F2 coordinates, shifting /ɛ/ toward the outside of the vowel space (ɛ-to-out perturbations). We applied three perturbation magnitudes in each perturbation direction: 0.5-, 1.0-, and 1.5-times participant-specific ɛ–æ distance. **(B)** In this panel, we illustrate the procedure for the calculation of corrective and adaptive responses. We defined adaptive responses as formant changes in early time points (0–100 ms) of the vowel in the unperturbed trial (n+1) after a perturbed trial (n) relative to the unperturbed trial (n−1) before the perturbed trial. We defined corrective responses as the difference between formant changes in the late time points (300–400 ms) of the vowel in a perturbed trial relative to the early time points (0–100 ms) of the vowel in the perturbed trial. The spectrograms in Panel **(B)** correspond to a generic production of /ɛ/ in the context of /hɛp/, used solely for illustration. In these spectrograms, time is displayed on the *x*-axis, frequency on the *y*-axis, and color (or intensity) represents the amplitude.

Participants completed seven blocks of compensation tasks in each experimental session, with a brief break between succeeding blocks. The overall design of the compensation task was similar to the pre-test task, and each block lasted about 7 min. Each block consisted of 120 trials, including 108 word-reading and 12 sentence-reading trials. These trials were distributed such that after every 18 word-reading trials, there were two sentence-reading trials. Participants produced /hɛp/, /hɛd/, or /hɛk/ in word-reading trials and produced a randomly selected sentence from the Harvard word list in sentence-reading trials. The rationale for including sentence-reading trials was threefold: (1) to provide a change in task to mitigate participant fatigue and boredom; (2) to serve as a washout period between trials and minimize long-lasting carry-over effects from the auditory perturbations; and (3) to maintain methodological consistency with the procedure employed in our previous study (Daliri et al., 2020). Participants always received unperturbed auditory feedback while speaking in the sentence-reading trials. However, during word-reading trials, participants received either (1) normal, unperturbed auditory feedback (unperturbed trials; 90 trials per block) or (2) formant-perturbed auditory feedback (perturbed trials; 18 trials per block). The perturbed trials were randomly presented, such that there were at least two unperturbed trials before each perturbed trial. Across all participants, there was an average of five unperturbed trials before each perturbed trial. In each perturbed trial, participants received one of the six participant-specific perturbation configurations: two perturbation directions (ɛ-to-æ and ɛ-to-out ) and three perturbation magnitudes (0.5, 1.0, and 1.5 ɛ–æ distance). In ɛ-to-æ perturbations, F1 and F2 of /ɛ/ were shifted along the ɛ–æ angle toward /æ/ in the F1-F2 coordinates (a concurrent F1-increase and F2-decrease). In ɛ-to-out perturbation, F1 and F2 of /ɛ/ were shifted along a line perpendicular to the ɛ-æ line and toward the outside of the vowel space in the F1-F2 coordinates (a concurrent F1-increase and F2-increase). The perturbation magnitudes were 0.5, 1.0, and 1.5 participant-specific ɛ–æ distance. Overall, there were 42 perturbed trials for each condition, defined by the combination of perturbation magnitude and direction. Figure 1A illustrates the perturbation configurations.

### Data analysis

The data analysis consisted of several steps using custom-written MATLAB scripts. First, an experimenter, blinded from the perturbation status, inspected the spectrograms of each trial to exclude gross formant-tracking errors and mispronunciations. The experimenter also manually selected the onset and offset of the vowel for each production. Second, we used the vowel onset and offset data to extract formant trajectories of each trial. We then calculated participant, experimental session, and word-specific “time-normalized” formant trajectories using unperturbed trials. Because the perturbation can influence the unperturbed trial immediately after a perturbed trial (Hantzsch et al., 2022), we did not include those unperturbed trials in the calculation of the time-normalized formant trajectories. For this purpose, we resampled formant trajectories from unperturbed trials at 100 points (regardless of their length) and averaged the resampled trajectories. This procedure aimed to accurately estimate the overall formant trajectories, including the consonant-vowel and vowel-consonant formant transitions. Third, for each trial, we interpolated the time-normalized trajectory to match its duration to the trial’s duration and then subtracted the interpolated time-normalized trajectories from the formant trajectories in the trial. This procedure effectively removed formant transitions due to the consonants at the beginning and end of the vowel (on a participant, session, and word-specific basis). Fourth, we extracted the F1 and F2 trajectories from the first 400 ms of each vowel production and excluded trials with durations less than 400 ms. Overall, we excluded 2.7% (SD = 4.4%) of all trials, and the number of excluded trials was similar across both experimental sessions (*p* = 0.516). Fifth, we calculated F1- and F2-adaptive responses by averaging formant changes over the 0–100 ms window. More specifically, we defined adaptive responses as formant changes in the unperturbed trial following a perturbed trial, relative to the unperturbed trial preceding the perturbed trial (Figure 1B). We also calculated F1 and F2 corrective responses by calculating the difference between formant changes in the 300–400 ms window relative to formant changes in the 0–100 ms window (Figure 1B). We normalized the magnitudes of the adaptive and corrective responses based on the participant-specific perturbation magnitudes. Note that we chose to analyze the F1 and F2 responses separately instead of calculating a projected response in the opposite direction of the perturbation (e.g., Daliri and Dittman, 2019) because (1) we applied perturbations in two different directions and (2) there was a large between-participant variability in the angle of responses to perturbations. Finally, we calculated the angles of the corrective and adaptive responses in the F1-F2 coordinates using the F1 and F2 responses. Because we were interested in the direction of responses relative to the ɛ-ɪ line (i.e., whether participants changed their formants toward their /ɪ/), we subtracted the response angles from the participant-specific ɛ-ɪ angle. We entered the angle and amplitude of F1 and F2 corrective responses, and the angle and amplitude of F1 and F2 adaptive responses, as dependent variables into the statistical analysis.

### Statistical analysis

We conducted all statistical analyses using *R* version 4.3.3 (R Core Team, 2021) in the *RStudio* environment version 2023.12.1 (RStudio Team, 2020). We used the *lme4* package (version 1.1.35.1) to fit a linear mixed-effects model (Bates et al., 2015) for each dependent variable, except the angles of corrective and adaptive responses (which required circular statistics). The original factorial design that included seven exposure blocks was too complex for the linear mixed-effects models, leading to non-convergence and singular fits. Note that each experimental session contained seven blocks of one perturbation direction, and each block of a given perturbation direction contained six repetitions of each of the three perturbation magnitudes. To ensure a statistically stable and parsimonious model while focusing on the core hypothesis of performance change with exposure, we collapsed the seven blocks for each perturbation direction (collected in one experimental session) into two levels (first and second halves; each half containing 3.5 blocks). This data reorganization allowed us to successfully fit a simplified linear mixed-effects model to each dependent variable. In these statistical models, we used perturbation direction (2 levels; ɛ-to-æ and ɛ-to-out ), perturbation magnitude (3 levels; 0.5, 1, 1.5 ɛ-æ distance), and exposure (2 levels; first half and second half) as fixed effects, and participant as a random intercept. We initially attempted to fit the maximal random effects structure (e.g., random slopes for the direction × magnitude × exposure interaction); however, this structure consistently led to non-convergence or singular fits, even after implementing a systematic step-down approach (Barr et al., 2013). Consequently, to achieve a statistically stable and parsimonious model, we specified a random-intercept-only structure, which accounts for baseline differences among participants and prevents over-parameterization. We confirmed model assumptions, including homoscedasticity and normality of residuals, by inspecting diagnostic plots (Harrison et al., 2018). We then obtained statistical significance of the fixed effects in the statistical model using *LmerTest* package (version 3.1.3) with Satterthwaite’s approximation method for estimating the degrees of freedom (Kuznetsova et al., 2017). We used the *emmeans* package (version 1.8.7) to conduct *post hoc* comparisons among levels of statistically significant fixed effects and to analyze statistically significant interactions (Lenth, 2019). We used the emmeans R package to estimate marginal means and perform these contrasts, applying the Kenward-Roger method to adjust standard errors and degrees of freedom. We corrected the *p*-values for multiple comparisons using the False Discovery Rate (FDR) method for all comparisons conducted within each model. Using the psych package (version 2.4.1), we assessed relationships among variables to calculate Pearson correlation coefficients (Revelle, 2018).

Given the inherent circularity (periodicity) of the corrective and adaptive response angles, we used the CircStats package (Lund and Agostinelli, 2025) to ensure the appropriate application of circular statistics. Based on an initial visual inspection, which suggested that the angles of response were consistent across different perturbation magnitudes and exposure, and to simplify subsequent analyses, we consolidated the data by calculating the circular mean of the response angles across these factors. To determine if the circular mean and angular deviation differed significantly between the two perturbation directions, we employed a non-parametric permutation test (Zar, 2010). This procedure involved randomly shuffling the direction labels (ɛ-to-æ and ɛ-to-out ) of the data points 10,000 times. For each shuffled iteration, we computed the difference in circular means and standard deviations between the two randomized conditions, thereby generating a null distribution for each statistic. The final *p*-value was then calculated as the proportion of null distribution differences that were equal to or more extreme than the observed difference in the original data.

## Results

**FIGURE 2:**
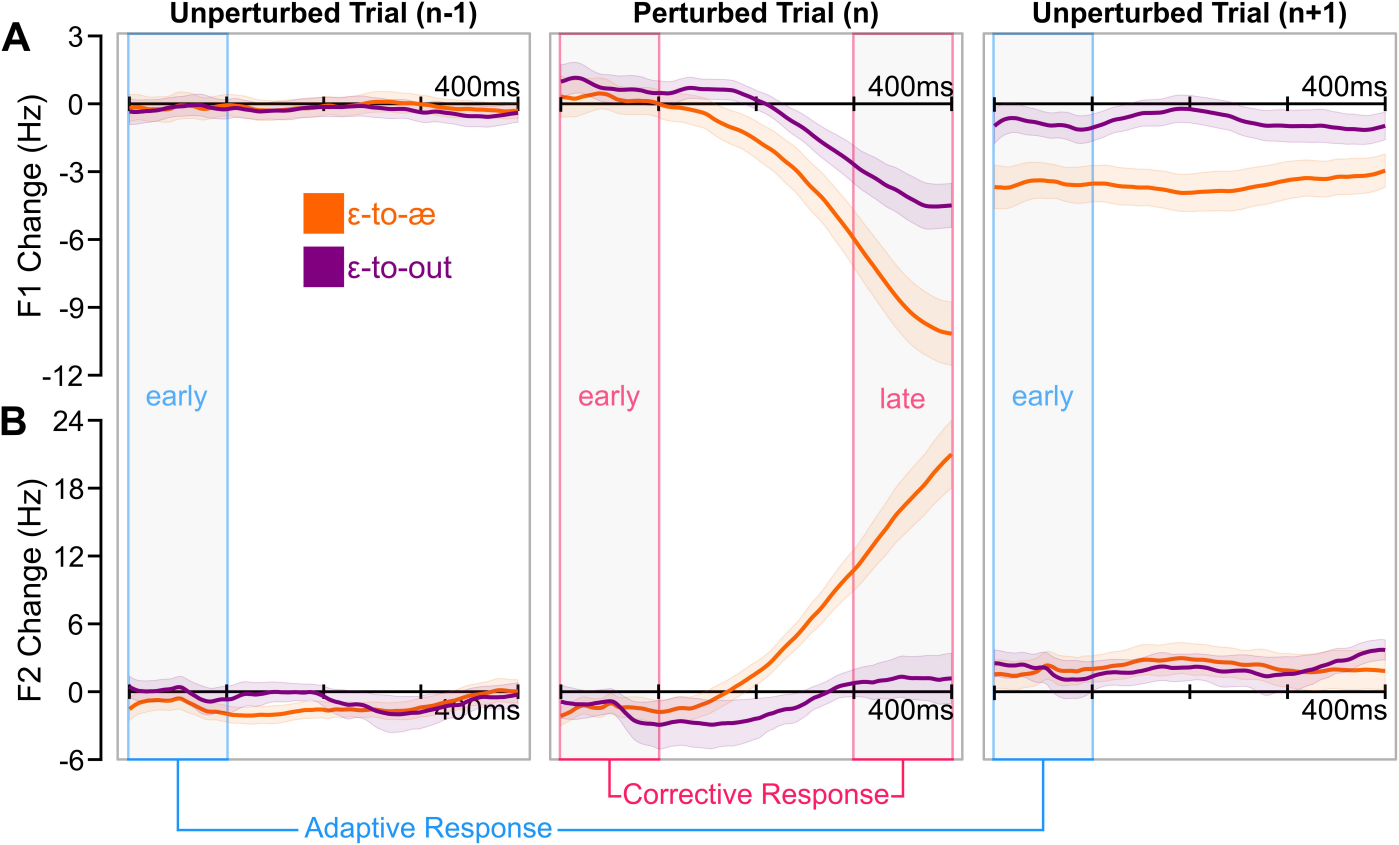
The group-average trajectories (averaged across perturbation magnitudes and exposure) of F1 and F2 change for perturbed trials (trial n) and unperturbed trials immediately before (trial n–1) and unperturbed trials immediately after (trial n+1) the perturbed trials are shown in panels **(A,B)**, respectively. Colored shaded areas represent the standard errors of the mean. For each formant, adaptive responses were defined as the change at early time points in the unperturbed trial following a perturbation (n+1) relative to the unperturbed trial preceding it (n−1). Corrective responses were defined as the difference between the late and early formant values within the perturbed trial (n).

**FIGURE 3:**
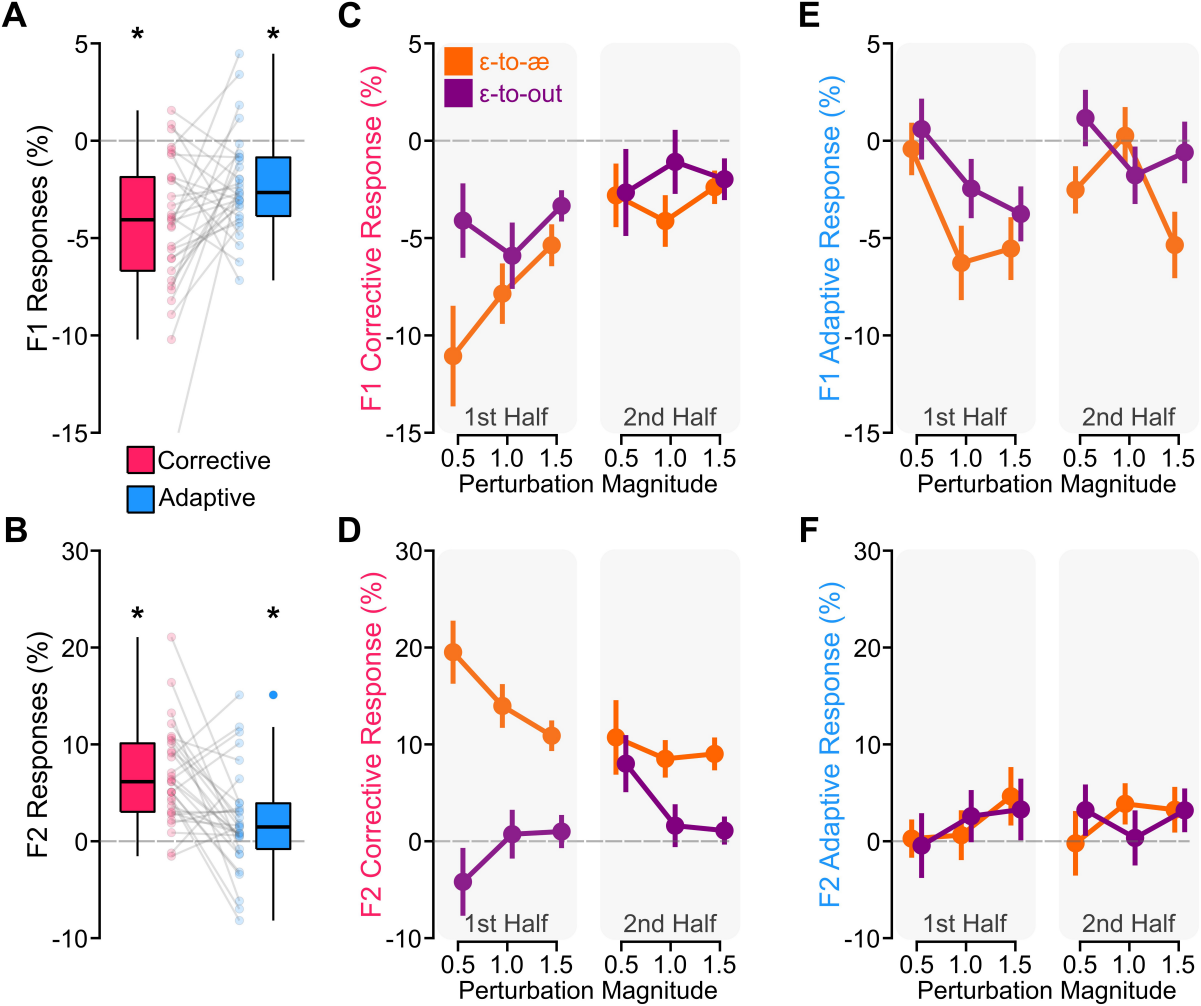
**(A,B)** show the distributions of individual data for F1 and F2 corrective and adaptive responses (averaged across all conditions). Our analysis showed that participants produced statistically significant F1 corrective and adaptive responses **(A)** and F2 corrective and adaptive responses **(B)** to compensate for errors induced by the perturbations (asterisks correspond to *p* < 0.05). **(C,D)** show the group-average results for F1 and F2 corrective responses in all perturbation conditions. Both F1 and F2 corrective responses were larger for ɛ-to-æ perturbations and proportionally larger for small perturbations in the first half of perturbed trials. **(E,F)** show the group-average results for F1 and F2 adaptive responses in all perturbation conditions. The F1 adaptive responses were larger for ɛ-to-æ perturbations. Error bars in panels **(C–F)** correspond to the standard errors of the mean.

We extracted and processed (see Section Data analysis) the first- and second-formant trajectories from the first 400 ms of the vowel in each production. Figure 2 shows the group-average formant changes in response to perturbations in the ɛ-to-æ and ɛ-to-out directions for the unperturbed trials before perturbations (Trial n−1), the perturbed trials (Trial n), and the unperturbed trials after the perturbations (Trial n+1). Then, to examine the effects of perturbations on feedback and feedforward systems, we calculated within-trial corrective responses and between-trial adaptive responses, respectively. To calculate corrective responses in perturbed trials, we determined the difference between formant values in the late (300–400 ms) and early (0–100 ms) intervals of the vowel. We estimated adaptive responses in unperturbed trials by calculating formant changes in the early interval of the post-perturbation trial relative to the early interval of the pre-perturbation trial. Figures 3A, B shows the distributions of F1 and F2 corrective responses and F1 and F2 adaptive responses (averaged across all perturbation directions, perturbation magnitudes, and exposure). We conducted a set of *a priori* one-sample *t*-tests to examine whether participants produced statistically significant corrective and adaptive responses (averaged across all perturbation directions, perturbation magnitudes, and exposure). In other words, these *a priori* tests examined whether responses differed from zero (i.e., no response). Our analysis showed that F1 corrective responses [*t*(29) = −6.422, *p* < 0.001], F2 corrective responses [*t*(29) = 6.867, *p* < 0.001], F1 adaptive responses [*t*(29) = −4.358, *p* < 0.001], and F2 adaptive responses [*t*(29) = 2.184, *p* = 0.038] were statistically different from zero (i.e., no response). In the following sections, we examine the effects of perturbation direction, perturbation magnitude, and exposure on the amplitude and angle of corrective and adaptive responses.

### The impact of perturbation direction, magnitude, and exposure on the amplitude of corrective and adaptive responses

#### Corrective responses

Figures 3C, D shows the group-average F1 and F2 corrective responses in all conditions. Our analysis of F1 corrective responses revealed statistically significant main effects of perturbation direction, *F*(1, 7041.5) = 14.884, *p* < 0.001, and exposure, *F*(1, 7032.8) = 36.708, *p* < 0.001. These main effects were modified by the statistically significant direction × magnitude × exposure interaction, *F*(2, 7032.5) = 3.221, *p* = 0.039. As shown in Figure 3C, this interaction indicated that F1 corrective responses to ɛ-to-æ perturbations were larger than responses to ɛ-to-out perturbations in the first half (*p* < 0.001), and this difference was the largest for the 0.5 magnitude (*p* < 0.004). However, F1 corrective responses to ɛ-to-æ perturbations were similar to responses to ɛ-to-out perturbations in the second half. Additionally, F1 corrective responses to 0.5 perturbation magnitude in the first half of ɛ-to-æ perturbations were larger than the responses to 0.5 perturbation magnitude in the second half of ɛ-to-æ perturbations. We examined F1 values during the initial 100 ms of the vowel in perturbed trials from the first and second halves to determine if the reduction in responses over time was due to the development of an adaptive response. This analysis did not reveal a statistically significant difference in formant values between the two halves (*p* > 0.05), suggesting that the change in corrective responses may not be driven by adaptation. The main effect of perturbation magnitude (*p* = 0.092), the direction × magnitude interaction (*p* = 0.279), the direction × exposure interaction (*p* = 0.056), and the magnitude × exposure interaction (*p* = 0.273) were not statistically significant.

Our analysis of F2 corrective responses revealed a statistically significant main effect of perturbation direction, *F*(1, 7042.0) = 107.487, *p* < 0.001. We also found statistically significant direction × exposure interaction, *F*(1, 7032.7) = 16.974, *p* < 0.001, and the direction × magnitude × exposure interaction, *F*(2, 7031.6) = 6.260, *p* = 0.002. As shown in Figure 3D, this interaction indicated that F2 corrective responses to ɛ-to-æ perturbations were generally larger than responses to ɛ-to-out perturbations in both halves (*p* < 0.001); this difference was the largest for the 0.5 magnitude in the first half (*p* = 0.005). Comparing responses in the first half showed that the F2 corrective responses to ɛ-to-æ perturbations were larger than the responses to ɛ-to-out perturbations (*p* < 0.001 for all perturbation magnitudes). However, in the second half, F2 corrective responses to ɛ-to-æ perturbations were larger than the responses to ɛ-to-out perturbations for 1.0 (*p* = 0.005) and 1.5 (*p* = 0.002) perturbation magnitudes but not for 0.5 perturbation magnitude. Similar to the analysis of F1 corrective responses, we examined F2 values during the initial 100 ms of the vowel in perturbed trials from the first and second halves to determine if the reduction in responses over time was due to the development of an adaptive response. This analysis did not reveal a statistically significant difference between the formants in the two halves (*p* > 0.05). The main effect of perturbation magnitude (*p* = 0.052), the main effect of exposure (*p* = 0.566), the direction × magnitude interaction (*p* = 0.406), and the magnitude × exposure interaction (*p* = 0.369) were not statistically significant.

#### Adaptive responses

Figures 3E, F shows the group-average results for F1 and F2 adaptive responses in all conditions. Examining F1 adaptive responses, we found statistically significant main effects of perturbation direction, *F*(1, 6863.0) = 4.026, *p* = 0.044, and magnitude, *F*(2, 6836.4) = 3.520, *p* = 0.029. These significant effects indicated that the F1 adaptive responses were larger for ɛ-to-æ perturbations, and responses to the perturbation magnitude of 0.5 were smaller than responses to the perturbation magnitude of 1.5 (*p* = 0.026). The main effect of exposure (*p* = 0.219), the direction × magnitude interaction (*p* = 0.634), the direction × exposure interaction (*p* = 0.813), the magnitude × exposure interaction (*p* = 0.261), and the direction × magnitude × exposure interaction (*p* = 0.149) were not statistically significant.

Our analysis of F2 adaptive responses did not reveal statistically significant main effect of direction (*p* = 0.894), main effect of magnitude (*p* = 0.333), main effect of exposure (*p* = 0.538), direction × magnitude interaction (*p* = 0.864), direction × exposure interaction (*p* = 0.576), magnitude × exposure interaction (*p* = 0.601), and direction × magnitude × exposure interaction (*p* = 0.323). Note that the average F2 adaptive response was 1.926% (*SD* = 4.832), and it was statistically significantly different from zero (*p* = 0.038).

#### Relationship between corrective and adaptive responses

To assess whether the feedback and feedforward control systems exhibited similar responses to errors, we examined the correlation between their respective behavioral measures (corrective and adaptive responses). We calculated the Pearson correlation coefficient between corrective and adaptive responses across all experimental conditions. The results indicated no statistically significant correlation coefficients for either the F1 or F2 responses in any condition.

### Examining the direction of corrective and adaptive responses

**FIGURE 4:**
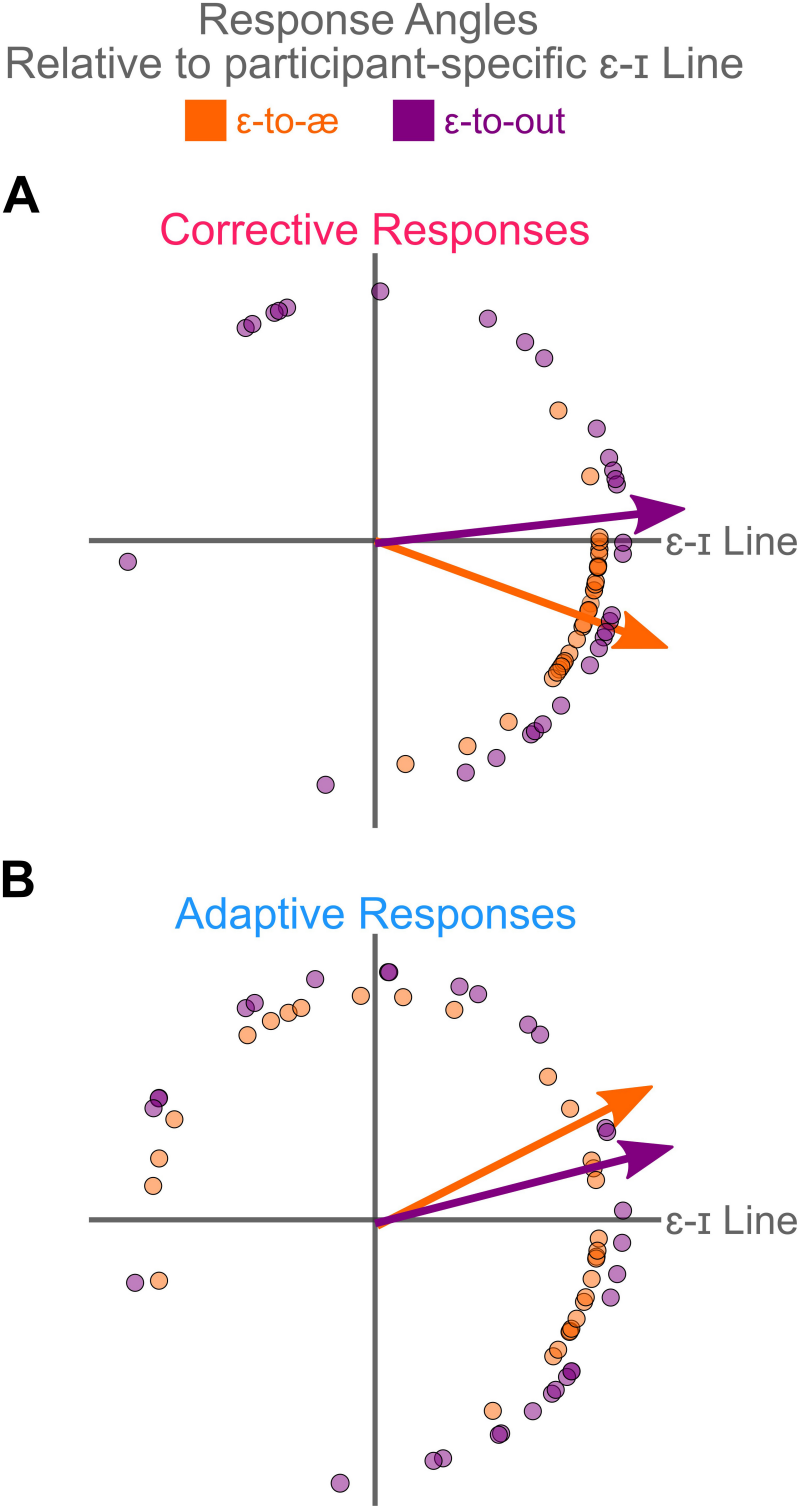
**(A,B)** illustrate the overall directionality of corrective and adaptive responses to the ɛ-to-æ and ɛ-to-out perturbations (response magnitudes are not shown in this figure). All response angles are calculated relative to the participant-specific ɛ-ɪ angle, which is shown along the positive *x*-axis (the angle zero). Regardless of the perturbation direction, participants produced corrective and adaptive responses that were generally aligned with the ɛ-ɪ line. Although the angle of the corrective responses to ɛ-to-æ perturbations was slightly different from the angles of corrective responses to ɛ-to-out perturbations, this pattern was not evident for the angles of adaptive responses. The arrows in panels **(A,B)** show the average response angle for each perturbation direction.

To examine the direction of corrective and adaptive responses, we first calculated the angle of responses in the F1-F2 coordinates. We then calculated the difference between the average response angle in each condition and the participant-specific ɛ-ɪ line angle. The rationale for averaging responses across trials in each condition and then calculating the angle was to obtain a more robust estimate of the angle. As shown in Figures 4A, B, the overall directions of the corrective and adaptive responses (arrows in A and B) to both ɛ-to-æ and ɛ-to-out perturbations were generally along the ɛ-ɪ direction.

As shown in Figure 4A, the mean angles of corrective responses to both perturbation directions were generally along the participant-specific ɛ-ɪ direction. Our analysis revealed that the angles of corrective responses to ɛ-to-æ perturbations (circular mean = −19.9 degrees relative to participant-specific ɛ-ɪ angle) were different (*p* = 0.027) from the angles of corrective responses to ɛ-to-out perturbations (circular mean = 4.5 degrees relative to participant-specific ɛ-ɪ angle). Additionally, we calculated angular deviation to examine the between-participant variability in response angles. The angular deviation is bounded (0−√2) and indicates whether the samples point to the same direction (angular deviation of zero) or they are spread out evenly around the circle (angular deviation of √2) (Lund and Agostinelli, 2025). Our analysis of the angular deviation of corrective responses revealed that the corrective responses to ɛ-to-out perturbations (angular deviation = 1.033) were more spread (*p* < 0.001) than the corrective responses to ɛ-to-æ perturbations (angular deviation = 0.378), indicating a larger between-participant variability in corrective responses to ɛ-to-out perturbations.

As shown in Figure 4B, the mean angles of adaptive responses to both perturbation directions were along the participant-specific ɛ-ɪ direction. Our analysis showed that the angles of responses to ɛ-to-æ perturbations (circular mean = 25.5 degrees relative to participant-specific ɛ-ɪ angle) were similar (*p* = 0.364) to the angles of corrective responses to ɛ-to-out perturbations (circular mean = 15.1 degrees relative to participant-specific ɛ-ɪ angle). Our analysis of the angular deviation of adaptive responses revealed no significant difference (*p* = 0.220) between the adaptive responses to ɛ-to-out perturbations (angular deviation = 1.192) and adaptive responses to ɛ-to-æ perturbations (angular deviation = 1.081), indicating a similar between-participant variability in adaptive responses to ɛ-to-out and ɛ-to-æ perturbations.

## Discussion

The primary objectives of the present study were to examine the effects of exposure extent to perturbations and perturbation direction on corrective and adaptive responses in a task in which participants experience formant perturbations in randomly selected trials (i.e., compensation paradigm). For this purpose, we applied three perturbation magnitudes (0.5, 1.0, and 1.5 ɛ–æ distance) in two different directions: (1) ɛ-to-æ direction by concurrently increasing F1 and decreasing F2 in the F1-F2 coordinates, and (2) ɛ-to-out direction by concurrently increasing F1 and increasing F2 in the F1-F2 coordinates (shifting /ɛ/ toward the outside of the vowel space). We examined responses in the first and second halves of trials to determine the effect of exposures to perturbations. We estimated corrective responses (a measure of the feedback-dependent system) in perturbed trials by calculating changes in formants in 300–400 ms of the vowel relative to formants in 0–100 ms of the vowel. We estimated adaptive responses (a measure of the feedforward system) in the unperturbed trials by calculating changes in formants in 0–100 ms of the vowel in the post-perturbation trial relative to formants in 0–100 ms of the vowel in the pre-perturbation trial. Our analyses showed that (1) corrective responses were larger for ɛ-to-æ perturbations and proportionally larger for small perturbations in the first half of perturbed trials, (2) both corrective and adaptive responses were generally in the ɛ-ɪ direction, regardless of the perturbation directions, and (3) adaptive responses were measurable after a single exposure to perturbation, especially after large ɛ-to-æ perturbations.

Our findings of larger corrective responses to ɛ-to-æ perturbations and proportionally larger responses to small perturbations in the first half of perturbed trials are consistent with those of our previous study (Daliri et al., 2020). For example, our previous study showed that corrective responses decreased proportionally as the magnitude of perturbations increased, and they were largest for ɛ-to-æ perturbations. These findings suggest that the brain may assign higher weights to errors induced by small ɛ-to-æ perturbations and thus generate larger responses to the errors. As previous studies have suggested (Chao and Daliri, 2023; Daliri and Dittman, 2019; Franken et al., 2023; Subramaniam et al., 2018), the brain may evaluate smaller and more natural errors (such as those induced by ɛ-to-æ perturbations) as more likely to be generated by its movements. However, larger and less natural errors (such as those induced by ɛ-to-out perturbations) are more likely to be generated by external sources, and the brain may assign a lower weight to them and consequently respond less to them (Christensen and Grünbaum, 2018; Haggard, 2017). Another potential explanation for these findings is that the brain’s relative reliance on somatosensory and auditory feedback may change when encountering different types of errors (Feng et al., 2011; Lametti et al., 2012). For example, when auditory errors become large or unnatural, the brain may rely more on somatosensory feedback for speech production and respond less to auditory error signals. It should be noted that these explanations are not necessarily mutually exclusive. Overall, our findings suggest that the brain evaluates small, natural errors differently from large, unnatural errors and responds to them differently.

In our previous study (Daliri et al., 2020), we applied simultaneous F1 and F2 perturbations along participant-specific ɛ–æ line and F1 perturbation only. Our results showed that regardless of the perturbation directions, participants’ corrective responses were along the participant-specific ɛ-ɪ line (i.e., participants changed their F1 and F2 to correct for both F1-F2 and F1 perturbations). Because both perturbations had similar perceptual consequences, it remained unclear whether the perceptual similarity of the perturbation had contributed to the similarity in response direction. Therefore, one objective of the current study was to determine the direction of corrective responses when auditory perturbations are orthogonally oriented (ɛ-to-æ vs. ɛ-to-out directions) so that their perceptual similarity is maximally reduced. In the present study, we found that corrective (and adaptive) responses were generally in the participant-specific ɛ-ɪ direction for ɛ-to-æ and ɛ-to-out perturbation directions. As a potential explanation for this finding, we speculate that the speech motor system potentially prefers generating movements that are acoustically aligned with the highly familiar, cardinal ɪ-ɛ-æ line in the formant space. We hypothesize that when the speech motor system detects an error induced by the novel ɛ-to-out perturbation, it defaults to using movement solutions along this ɪ-ɛ-æ line, even though responding along this axis may not perfectly compensate for the ɛ-to-out error. While responding along the ɪ-ɛ-æ line effectively compensates for ɛ-to-æ errors, it does not compensate for ɛ-to-out . This speculation is partially supported by the observation that there was greater individual variability in the response direction to the ɛ-to-out perturbation, suggesting that different individuals use different solutions (see Figure 4A). Notably, this directional bias is not attributable to a carry-over effect from experiencing ɛ-to-æ perturbations. This conclusion is supported by our experimental design, which included (at least) a 7-days separation between sessions to mitigate short-term effects, and by systematic counterbalancing of perturbation order across participants, which effectively eliminated session-order effects. Overall, we argue that the observed response pattern for ɛ-to-out indicates that the speech motor system’s biases in selecting movement solutions may influence error compensation, leading to responses that prioritize preferred movement solutions even when they do not entirely correct errors (e.g., responding in the ɛ-ɪ direction for ɛ-to-out perturbations).

One of the intriguing findings of the current study is the significant interactions involving the exposure effect for the corrective responses. Specifically, in comparing corrective responses in the first half versus the second half of trials, we found that corrective responses for ɛ-to-æ perturbations were proportionally larger for small perturbations in the first half of perturbed trials. In other words, with more exposure to perturbed trials, participants produced smaller corrective responses. Importantly, our *post hoc* analyses indicated that this reduction was not due to adaptation to the perturbations. This pattern of decreasing corrective responses across trials may resemble the pattern observed in adaptation paradigms (Daliri, 2021; Kearney et al., 2020). However, there are fundamental differences between these two patterns. In adaptation studies, participants experience perturbations across consecutive trials and gradually develop adaptive responses to compensate for the induced errors. Therefore, the errors gradually become smaller, leading to smaller corrective responses. In contrast, in a compensation paradigm, the perturbations are not repeated across consecutive trials to minimize the development of an adaptive response, leading to similar errors across perturbed trials (and presumably similar corrective responses). We speculate that as participants experience more unexpected perturbations, they may begin to evaluate such errors differently–for example, assigning them more strongly to external sources–and thus produce smaller corrective responses to compensate for them (Berniker and Körding, 2008). The failure to observe a reduction in corrective response magnitude for the ɛ-to-out perturbations, in contrast to the ɛ-to-æ perturbations, may reflect the already small scale of responses to ɛ-to-out perturbations, making a statistically significant decrease difficult to detect. However, a methodological limitation inherent to compensation paradigms–the necessity of repeated error exposure–cannot be ignored. Prolonged exposure to perturbations throughout the session, while leading to more robust corrective responses, can potentially lead to participant fatigue, cognitive habituation, or reduced attention. Because we did not explicitly measure these cognitive factors and their impact on corrective responses is not well understood, we cannot definitively rule out a partial contribution of these factors to the observed attenuation of corrective responses. Consequently, for future compensation studies, we recommend limiting the total number of perturbed trials; greater exposure to error may, in practice, diminish the magnitude of elicited corrective responses.

Finally, our finding of adaptive responses following a single perturbed trial (especially after large ɛ-to-æ perturbations) was consistent with a previous study (Hantzsch et al., 2022). By pooling data from six studies, Hantzsch et al. (2022) showed that participants develop small adaptive responses after experiencing one perturbed trial. More specifically, they reported that F1 adaptive responses (measured at 0–100 ms of the vowel) in trials after an upward F1 shift differed from F1 adaptive responses in trials after a downward F1 shift. Although this method of estimating adaptive responses differs from that used in the present study, our overall finding of F1 adaptive responses in post-perturbation trials is consistent with their results. It should be noted that in our previous study (Daliri et al., 2020), we compared adaptive responses in the trial before a perturbed trial with responses in the trial after the perturbed trial. However, we failed to find statistically significant adaptive responses. Similarly, Parrell et al. (2017) examined changes in F1 during the first 50 ms of the vowel in post-perturbation trials and failed to find significant F1 adaptive responses. We believe previous studies’ failure to find adaptive responses is related to methodological issues such as (1) the number of unperturbed trials before a perturbed trial, (2) adjusting formants based on formants of unperturbed trials, and (3) formant transition at the onset of vowels. A higher number of unperturbed trials before a perturbed trial would significantly reduce the effect of the previous perturbation. Therefore, when a post-perturbation trial is compared with a pre-perturbation trial, the effect of the perturbation is more salient. In compensation paradigms, the average formant value from unperturbed trials is subtracted from the formant values across all trials, resulting in formant changes close to zero in unperturbed trials. However, this analysis technique reduces the salience of adaptive responses in post-perturbation trials. Therefore, to increase sensitivity for detecting adaptive responses, we did not use unperturbed trials immediately after perturbed trials in the subtraction procedure (for adjusting formant transitions), as these trials may contain evidence of adaptive responses. Lastly, the consonant-vowel formant transition at the beginning of the vowel may also reduce the likelihood of finding adaptive responses. This issue is critical, as the initial formant transitions in the post- and pre-perturbation trials may differ substantially if the initial consonants in the two trials differ. Therefore, it is important to adjust the formants on a word-specific basis. Finally, despite a significant overall adaptive response (across all conditions), the magnitude-specific findings were unexpected: the adaptive response to the smallest perturbation (0.5) was non-significant, and adaptive responses did not scale proportionally with increasing perturbation magnitudes. We speculate that this non-significance reflects a limitation of the single-trial measurement protocol, where the adaptive response elicited by a minimal 0.5 perturbation is simply too small for robust detection. Alternatively, the scaling relationship between adaptive response and perturbation magnitude may be genuinely non-uniform. Future studies must systematically vary perturbation magnitude to map how adaptive amplitude scales with error magnitude. Overall, our findings of adaptive responses in post-perturbation trials are consistent with previous studies, and they provide complementary evidence for “one-shot adaptation” in speech movements as described by Hantzsch et al. (2022) and “one-shot adaptation” in limb movements (e.g., Smith and Shadmehr, 2005).

In summary, we used a compensation paradigm (in which auditory perturbations were applied in random trials) to estimate corrective and adaptive responses to perturbations across different directions and magnitudes. We found that (1) corrective responses were larger in responses to perturbation that shifted /ɛ/ toward /æ/, (2) corrective responses were proportionally larger for small perturbations in the first half of perturbed trials, (3) both corrective and adaptive responses were in the ɛ-ɪ direction regardless of the perturbation directions, and (4) adaptive responses were measurable after a single exposure to perturbation. These findings suggest that the brain evaluates small, natural-sounding errors (induced by ɛ-to-æ perturbations) differently from large, unnatural errors, and responds to them differently. The brain’s biases in selecting movement solutions may also influence its error compensation, leading to responses that prioritize preferred movement solutions even when they do not entirely correct errors (e.g., responding in the ɛ-ɪ direction for ɛ-to-out perturbations). Additionally, our results suggest that with greater exposure to unexpected perturbations, the brain evaluates them differently; it may assign the errors to external sources more strongly, leading to smaller corrective responses.

## Data Availability

The datasets presented in this article are not readily available because the data set collected for the current study is only available on reasonable requests. Requests to access the datasets should be directed to Ayoub.Daliri@asu.edu.

## References

[B1] ASHA (1997). *Guidelines for audiologic screening: Panel on audiologic assessment.* Rockville, MA: American Speech-Language-Hearing Association.

[B2] Barr D. J. Levy R. Scheepers C. Tily H. J. (2013). Random effects structure for confirmatory hypothesis testing: Keep it maximal. *J. Mem. Lang.* 68 255–278. 10.1016/j.jml.2012.11.001 24403724 PMC3881361

[B3] Basciano A. Chao S.-C. Gomez A. Daliri A. Rogalsky C. (2025). Individuals with aphasia generate larger adaptive and corrective responses to suddenly introduced auditory perturbations. *Front. Hum. Neurosci.* 19:1672114. 10.3389/fnhum.2025.1672114 41394943 PMC12696673

[B4] Bates D. Mächler M. Bolker B. M. Walker S. C. (2015). Fitting linear mixed-effects models using lme4. *J. Stat. Softw.* 67 1–48. 10.18637/jss.v067.i01

[B5] Behroozmand R. Khoshhal Mollasaraei Z. Nejati V. Daliri A. Fridriksson J. (2025). Vocal and articulatory speech control deficits in individuals with post-stroke aphasia. *Sci. Rep.* 15:13350. 10.1038/s41598-025-96040-4 40246982 PMC12006427

[B6] Berniker M. Körding K. P. (2008). Estimating the sources of motor errors for adaptation and generalization. *Nat. Neurosci.* 11 1454–1461. 10.1038/nn.2229 19011624 PMC2707921

[B7] Burnett T. A. Senner J. E. Larson C. R. (1997). Voice F0 responses to pitch-shifted auditory feedback: A preliminary study. *J. Voice* 11 202–211. 10.1016/S0892-1997(97)80079-3 9181544

[B8] Cai S. (2015). *Audapter.* Available online at: https://github.com/shanqing-cai/audapter_matlab (accessed August 16, 2022).

[B9] Cai S. Beal D. S. Ghosh S. S. Guenther F. H. Perkell J. S. (2014). Impaired timing adjustments in response to time-varying auditory perturbation during connected speech production in persons who stutter. *Brain Lang.* 129 24–29. 10.1016/j.bandl.2014.01.002 24486601 PMC3947674

[B10] Chao S. Daliri A. (2023). Effects of gradual and sudden introduction of perturbations on adaptive responses to formant-shift and formant-clamp perturbations. *J. Speech Lang. Hear. Res.* 66 1588–1599. 10.1044/2023_JSLHR-21-00435 37059081 PMC10457088

[B11] Chao S. Ochoa D. Daliri A. (2019). Production variability and categorical perception of vowels are strongly linked. *Front. Hum. Neurosci.* 13:96. 10.3389/FNHUM.2019.00096 30967768 PMC6439354

[B12] Christensen M. S. Grünbaum T. (2018). Sense of agency for movements. *Consciousness Cogn.* 65 27–47. 10.1016/j.concog.2018.07.002 30007133

[B13] Daliri A. (2021). A computational model for estimating the speech motor system’s sensitivity to auditory prediction errors. *J. Speech Lang. Hear. Res.* 64 1841–1854. 10.1044/2021_JSLHR-20-00484 34043445 PMC8740760

[B14] Daliri A. Dittman J. (2019). Successful auditory motor adaptation requires task-relevant auditory errors. *J. Neurophysiol.* 122 552–562. 10.1152/jn.00662.2018 31215301

[B15] Daliri A. Max L. (2018). Stuttering adults’ lack of pre-speech auditory modulation normalizes when speaking with delayed auditory feedback. *Cortex* 99 55–68. 10.1016/j.cortex.2017.10.019 29169049 PMC5801108

[B16] Daliri A. Chao S. Fitzgerald L. C. (2020). Compensatory responses to formant perturbations proportionally decrease as perturbations increase. *J. Speech Lang. Hear. Res.* 63 3392–3407. 10.1044/2020_JSLHR-19-00422 32976078 PMC8060011

[B17] Daliri A. Honda S. Max L. (2025). Delayed auditory feedback increases speech production variability in typically fluent adults but has the opposite effect in stuttering adults. *Front. Hum. Neurosci.* 19:1628114. 10.3389/fnhum.2025.1628114 40949722 PMC12425929

[B18] Feng Y. Gracco V. L. Max L. (2011). Integration of auditory and somatosensory error signals in the neural control of speech movements. *J. Neurophysiol.* 106 667–679. 10.1152/jn.00638.2010 21562187 PMC3154803

[B19] Franken M. K. Hartsuiker R. J. Johansson P. Hall L. Lind A. (2023). Don’t blame yourself: Conscious source monitoring modulates feedback control during speech production. *Quart. J. Exp. Psychol.* 76 15–27. 10.1177/17470218221075632 35014590

[B20] Guenther F. H. (2016). *Neural control of speech.* Cambridge, MA: MIT Press.

[B21] Haggard P. (2017). Sense of agency in the human brain. *Nat. Rev. Neurosci.* 18 196–207. 10.1038/nrn.2017.14 28251993

[B22] Hantzsch L. Parrell B. Niziolek C. A. (2022). A single exposure to altered auditory feedback causes observable sensorimotor adaptation in speech. *eLife* 11:e73694. 10.7554/eLife.73694 35816163 PMC9302966

[B23] Harrison X. A. Donaldson L. Correa-Cano M. E. Evans J. Fisher D. N. Goodwin C. E. D. (2018). A brief introduction to mixed effects modelling and multi-model inference in ecology. *PeerJ* 6:e4794. 10.7717/peerj.4794 29844961 PMC5970551

[B24] Heinks-Maldonado T. H. Houde J. F. (2005). Compensatory responses to brief perturbations of speech amplitude. *Acoustics Res. Lett. Online* 6 131–137. 10.1121/1.1931747

[B25] Houde J. F. Jordan M. I. (1998). Sensorimotor adaptation in speech production. *Science* 279 1213–1216. 10.1126/science.279.5354.1213 9469813

[B26] Houde J. F. Nagarajan S. S. (2011). Speech production as state feedback control. *Front. Hum. Neurosci.* 5:82. 10.3389/fnhum.2011.00082 22046152 PMC3200525

[B27] Kearney E. Nieto-Castañón A. Weerathunge H. R. Falsini R. Daliri A. Abur D. (2020). A simple 3-parameter model for examining adaptation in speech and voice production. *Front. Psychol.* 10:2995. 10.3389/fpsyg.2019.02995 32038381 PMC6985569

[B28] Kim K. S. Wang H. Max L. (2020). It’s about time: Minimizing hardware and software latencies in speech research with real-time auditory feedback. *J. Speech Lang. Hear. Res.* 63 2522–2534. 10.1044/2020_JSLHR-19-00419 32640180 PMC7872729

[B29] Krakauer J. W. Hadjiosif A. M. Xu J. Wong A. L. Haith A. M. (2019). Motor learning. *Comprehensive Physiol.* 9 613–663. 10.1002/cphy.c170043 30873583

[B30] Kuznetsova A. Brockhoff P. B. Christensen R. H. B. (2017). lmerTest package: Tests in linear mixed effects models. *J. Stat. Softw.* 82 1–26. 10.18637/jss.v082.i13

[B31] Lametti D. R. Nasir S. M. Ostry D. J. (2012). Sensory preference in speech production revealed by simultaneous alteration of auditory and somatosensory feedback. *J. Neurosci.* 32 9351–9358. 10.1523/JNEUROSCI.0404-12.2012 22764242 PMC3404292

[B32] Lenth R. (2019). *emmeans: Estimated marginal means. R package.* Available online at: https://cran.r-project.org/package=emmeans (accessed May 12, 2025).

[B33] Lester-Smith R. A. Daliri A. Enos N. Abur D. Lupiani A. A. Letcher S. (2020). The relation of articulatory and vocal auditory-motor control in typical speakers. *J. Speech Lang. Hear. Res.* 63 3628–3642. 10.1044/2020_JSLHR-20-00192 33079610 PMC8582832

[B34] Lund U. Agostinelli C. (2025). *CircStats: Circular statistics, from “Topics in circular statistics” (2001).* Available online at: https://CRAN.R-project.org/package=CircStats (accessed May 12, 2025).

[B35] Maas E. Robin D. A. Hula S. N. Freedman S. E. Wulf G. Ballard K. J. (2008). Principles of motor learning in treatment of motor speech disorders. *Am. J. Speech Lang. Pathol.* 17 277–298. 10.1002/9781118660584.ese1594 18663111

[B36] McNamee D. Wolpert D. M. (2019). Internal models in biological control. *Annu. Rev. Control Robotics Autonomous Syst.* 2 339–364. 10.1146/annurev-control-060117-105206 31106294 PMC6520231

[B37] Merrikhi Y. Parsa M. Daliri A. (2025). An integrated approach to concurrently measure corrective and adaptive responses to auditory errors. *J. Speech Lang. Hear. Res.* 68 3748–3758. 10.1044/2025_jslhr-24-00572 40587266 PMC12384895

[B38] Nejati V. Daliri A. Behroozmand R. (2025). Neural mechanisms of articulatory motor speech deficit in post-stroke aphasia: An ERP study. *NeuroImage* 320:121483. 10.1016/j.neuroimage.2025.121483 41005583 PMC13090139

[B39] Parrell B. Agnew Z. K. Nagarajan S. S. Houde J. F. Ivry R. B. (2017). Impaired feedforward control and enhanced feedback control of speech in patients with cerebellar degeneration. *J. Neurosci.* 37 3363–3316. 10.1523/JNEUROSCI.3363-16.2017 28842410 PMC5607467

[B40] Parrell B. Houde J. F. (2019). Modeling the role of sensory feedback in speech motor control and learning. *J Speech Lang. Hear. Res.* 62 2963–2985. 10.1044/2019_JSLHR-S-CSMC7-18-0127 31465712 PMC6813034

[B41] Perkell J. S. (2012). Movement goals and feedback and feedforward control mechanisms in speech production. *J. Neurolinguistics* 25 382–407. 10.1016/j.jneuroling.2010.02.011 22661828 PMC3361736

[B42] Purcell D. W. Munhall K. G. (2006). Compensation following real-time manipulation of formants in isolated vowels. *J. Acoustical Soc. Am.* 119 2288–2297. 10.1121/1.2173514 16642842

[B43] R Core Team. (2021). *R: A language and environment for statistical computing.* Vienna: R Foundation for Statistical Computing.

[B44] Revelle W. (2018). *psych: Procedures for psychological, psychometric, and personality research.* Evanston, IL: Northwestern University.

[B45] RStudio Team. (2020). *RStudio: Integrated development for R.* Boston: RStudio, PBC.

[B46] Shadmehr R. Mussa-Ivaldi S. (2012). *Biological learning and control: How the brain builds representations, predicts events, and makes decisions.* Cambridge, MA: MIT Press.

[B47] Smith M. A. Shadmehr R. (2004). *Modulation of the rate of error-dependent learning by the statistical properties of the task.* Washington, DC: Society for Neuroscience.

[B48] Smith M. A. Shadmehr R. (2005). Intact ability to learn internal models of arm dynamics in Huntington’s disease but not cerebellar degeneration. *J. Neurophysiol.* 93 2809–2821. 10.1152/jn.00943.2004 15625094

[B49] Stepp C. E. Lester-Smith R. A. Abur D. Daliri A. Noordzij P. J. Lupiani A. A. (2017). Evidence for auditory-motor impairment in individuals with hyperfunctional voice disorders. *J. Speech Lang. Hear. Res.* 60 1545–1550. 10.1044/2017_JSLHR-S-16-0282 28590007 PMC5544411

[B50] Subramaniam K. Kothare H. Mizuiri D. Nagarajan S. S. Houde J. F. (2018). Reality monitoring and feedback control of speech production are related through self-agency. *Front. Hum. Neurosci.* 12:82. 10.3389/FNHUM.2018.00082/BIBTEX 29559903 PMC5845688

[B51] Terband H. Brenk F. van, Zee A. van D. der. (2014). Auditory feedback perturbation in children with developmental speech sound disorders. *J. Commun. Disord.* 51 64–77. 10.1016/j.jcomdis.2014.06.009 25127854

[B52] Tourville J. A. Reilly K. J. Guenther F. H. (2008). Neural mechanisms underlying auditory feedback control of speech. *NeuroImage* 39 1429–1443. 10.1016/j.neuroimage.2007.09.054 18035557 PMC3658624

[B53] Zar J. H. (2010). *Biostatistical analysis*, 5th Edn. Hoboken, NJ: Pearson Prentice Hall.

